# Secondary school students’ perspectives on their school food environments and their proposed solutions for improvement: Case study in Northern Benin

**DOI:** 10.1371/journal.pone.0361942

**Published:** 2026-11-03

**Authors:** Mélina Maureen Houndolo, Fifali Sam Ulrich Bodjrenou, Waliou Amoussa Hounkpatin, Fadilatou Chabi Yorouba, Hessede Mondoukpe Gbahougbola, Deo Grathias Yevedo Kossa, Irene Mitchodigni, Teresa Borelli, Celine Termote

**Affiliations:** 1 Bioversity International, Cotonou, Benin; 2 University of Abomey-Calavi, Faculty of Agricultural Sciences, Cotonou, Benin; 3 International Center for Tropical Agriculture (CIAT), Cotonou, Benin; 4 World Vegetable Center, Cotonou, Benin; 5 Bioversity International, Rome, Italy; 6 Bioversity International, Nairobi, Kenya; University of Georgia, UNITED STATES OF AMERICA

## Abstract

**Background:**

The food environment within and around schools plays a critical role in shaping secondary school students’ current and future eating behavior, food choices, and perceptions of healthy diets. However, little is known about how secondary school students in low-income settings perceive these environments and how such perceptions may influence their food practices.

**Methods:**

A qualitative study was conducted in the municipalities of Natitingou (urban) and Boukombe (rural), Northern Benin, to explore secondary school students’ perceptions of their school food environments. In each municipality, two focus group discussions were held: one with lower-secondary students (11–15 years), and one with upper-secondary students (14–19 years), ensuring equal gender representation. Discussions addressed the types of foods available within and around schools, students’ food preferences and perceptions, and suggestions for improving their food environments. Data were analyzed thematically.

**Results:**

Students reported access to a wide variety of foods within and near their schools, particularly in urban settings. However, most of these foods were energy-dense and high in sugar, while fruit and vegetables were rarely available. Participants perceived their school food environments as largely unhealthy, citing inadequate food hygiene practices and poor vendor sanitation as major concerns.

**Conclusion:**

Findings highlight the need to integrate secondary school students’ perspectives into efforts to improve school food environments in Benin. Policies promoting the availability of nutritious foods and strengthening hygiene standards in and around schools could support healthier dietary behaviors among students. Future research should examine how these environments and perceptions influence secondary school students’ long-term nutrition and health outcomes.

## Introduction

Health, nutrition, and education are interrelated elements that contribute to the development of young people and, in the long term, to the prosperity of society [1]. Improving the health and nutritional status of school-age children and adolescents is essential not only for their immediate well-being but also for future global development. Indeed, the prevalence of obesity among children and adolescents in the African Region is predicted to rise rapidly between 2020 and 2035, with girls in particular, seeing a major increase from 5−14 percent in sub-Saharan Africa [2]. Adolescence is a critical period, during which balanced nutrition and healthy eating habits must be established to prevent diseases and promote health in adulthood [3,4]. Several studies have emphasized concerns regarding the eating habits of young people, which are marked by insufficient fruit and vegetable intake, and a high consumption of processed foods [5–7].

Among the factors influencing nutritional health, the food environment plays a key role. It encompasses the physical(food outlets location, food availability…), economic (food prices), political, and sociocultural (cultural or religious food restriction, food preferences…) conditions that affect individual food choices and nutritional status [8–10]. Importantly, a distinction must be drawn between the objective food environment (i.e., the measurable physical, economic, and policy characteristics of food settings) and the perceived food environment (i.e., how individuals subjectively experience and interpret food availability, accessibility, affordability, and desirability). This distinction is critical because perceived food environments may influence dietary behaviors independently of objectively measured conditions [8,10,11]. Schools are important settings for promoting healthy eating because they influence food availability, food choices, and dietary behaviors during adolescence [12–14]. The school food environment encompasses the physical, economic, policy, and socio‑cultural contexts within and around school settings that influence what foods are available, accessible, and promoted, as well as how students interact with and perceive these environments to make decisions about acquiring, preparing, and consuming food [14–16]. The school food environment may influence students’ perceptions of healthy food choices, as they spend on average 6−9 hours per day at school [17]. Healthy school food environments can support healthier dietary behaviors and contribute to broader nutrition and Sustainable Development Goals such as SDG-2 (Zero hunger), and SDG-3 (Good health and well-being), as well as providing a unique opportunity for transitioning to planet-friendly policies with important co-benefits for child health and wider society [16,18,19].

In Benin, as in several developing countries, children and young people face major nutritional challenges, with alarming rates of obesity and overweight among these two groups [20]. The most recent study reported that 8.1 percent of school-going adolescents were overweight and 1.6 percent were obese indicating that excess weight is already a significant concern in urban and peri-urban adolescent populations [21]. Many secondary school students fail to consume adequate amounts of fruit and vegetables, despite recommendations from the Benin dietary guidelines (Double Nutritional Burden Pole) and the Food and Agriculture Organization [22].While a growing body of literature examines school food environments globally, most studies have focused on objectively measured food availability in high-income settings. Few have investigated how students themselves perceive their school food environment, particularly in food-insecure regions of sub-Saharan Africa where no formal feeding programs exist at the secondary level. This study extends Turner et al.’s food environment framework [10] by shifting the analytical lens from objective measurement to adolescents’ subjective perceptions, thereby foregrounding the personal dimension of the food environment in a context where it has rarely been examined. Examining these perspectives can provide practical insights to improve the availability and quality of foods in schools, directly supporting SDG targets 2.2 (ending malnutrition) and 3.4 (reducing diet-related non-communicable diseases). Indeed, the national school feeding program implemented in Benin [23], targets only elementary schools. At the secondary level, food provision relies entirely on informal vendors located in and around schools. Secondary school food environments also differ from primary school settings due to greater student autonomy, weaker supervision, increased purchasing power, stronger peer influence, and a more commercialized food offer, making adolescents more vulnerable to unhealthy dietary patterns [24,25]. Against this context, this study used a qualitative approach to explore students’ perceptions of food availability, diversity, and quality in and around schools, how they perceive these aspects of the school food environment in relation to their food choices, and the solutions they propose to improve the school food environment.

## Methods

### Study area

The study was conducted in northern Benin, in the department of Atacora in two municipalities, notably Boukombe (10.1833° N, 1.1000° E), representing rural settings and Natitingou (10.296353° N, 1.380690° E) representing an urban area. The department of Atacora is one of the departments in Benin most affected by malnutrition and food insecurity, with 36 percent of children experiencing stunted growth. The municipalities of Natitingou and Boukombe are among those with the highest prevalence of malnutrition and food insecurity in Atacora [26]. In these municipalities, approximately 32.9 percent of households experience insufficient food consumption, with more than two in ten households classified as having poor food consumption levels [27].

### Study design

The study was conducted from November to December 2022 in the northern part of Benin, specifically in Atacora department, and focused on two secondary schools in Boukombe (rural area) and Natitingou (urban area). In each municipality, the main secondary school identified as the one with the highest student enrollment was selected for the study. Selecting the largest school allowed us to capture a diverse range of student backgrounds and experiences, which is particularly important in qualitative research exploring perceptions. Due to resource and logistical constraints, it was not feasible to include multiple schools per municipality; focusing on the largest school ensured that we could obtain rich and varied data while maintaining depth of inquiry. In each selected school, two focus group discussions were conducted: One with the first lower secondary school (11–15 years of age) and the second with those from the upper secondary school (14–19 years of age).

### Study participants

Prior to the work, both schools obtained authorization from the Ministry of Education and the Departmental Directorate of Secondary, Technical, and Vocational Education, as well as consent from the school principals, teachers, and parents of the students involved.

Once the schools were selected, participants for the focus group discussions (FGDs) were recruited using two-stage sampling strategy combining purposive and random selection. In the first stage, purposive sampling was used to define the selection criteria and ensure representation across grade levels and genders. Specifically, grade levels were intentionally selected to capture a range of adolescent experiences within each school. In the second stage, within each selected grade level, students who met the inclusion criteria were listed, and participants were then randomly drawn from this list to minimize selection bias while maintaining gender balance. This combined approach ensured both the diversity needed for meaningful group discussions and the fairness of individual participant selection [28].

- Inclusion criteria

Students eligible for inclusion in this study met the following criteria:

- Enrollment in one of the selected schools.
- Enrollment in a grade level not involved in end-of-year exams.
- Consent form signed.
- Exclusion criteria

Students in exam classes were not included.

Two FGDs of approximately one hour were conducted in each municipality, separating students from the lower secondary school (11–15 years of age) and those from the upper secondary school (14–19 years of age) (Table 1). A total of 40 students has been included in this study. The number of FGDs was guided by the principle of data saturation, meaning that data collection continued until no new themes or insights emerged from subsequent discussions. Saturation was assessed iteratively during debriefing sessions after each FGD, where the research team compared newly emerging codes with those already identified. Evidence of saturation was determined when successive FGDs yielded no substantively new themes, sub‑themes beyond those already captured in the evolving codebook, indicating that the range of student perspectives had been adequately represented [29].

**Table 1:** Participants characteristics.

Schools	FGD	Age group	Age range (years)	Number of participants	Girls (n)	Boys (n)	Grade level
Boukombe	1	lower secondary school	11-15	10	5	5	6th-8th
	2	Upper secondary school	14-19	10	5	5	10th-11th
Natitingou	3	lower secondary school	11-15	10	5	5	6th-8th
	4	Upper secondary school	14-19	10	5	5	10th-11th

### Data collection

Focus group discussions (FGDs) were conducted with students to capture their perceptions of their school food environments using an interview guide. The interview guide was pre-tested with non-participating students, and feedback was used to refine wording and probes; it was also reviewed and validated by the multidisciplinary research team to ensure content relevance and clarity. This guide was conceptually informed by Turner et al.’s food environment framework [10]. The FGDs were conducted in both French and local languages spoken by participants (Ditammari in Boukombe and Waama in Natitingou), as these are the languages in which students feel most comfortable expressing themselves, thereby facilitating richer and more authentic responses. Local languages have been used mostly by the students for the name of foods. Each FGD was conducted by two members: one moderator and one note-taker. Each session lasted approximately 45–60 minutes and took place in a quiet, private room within the school premises to ensure confidentiality and minimize distractions. To avoid dominance bias technics like turn-taking prompts, targeted invitations to quieter participants, and gentle redirection of overly dominant speakers were used. The discussions focused on food availability in the school and its surroundings (1 km), as well as students’ perceptions of their school’s food environment and their proposed solutions for improvement. The sessions were audio-recorded with the students’ consent, and field notes were also taken.

### Data processing and analysis

The FGD audio recordings were transcribed verbatim in the original languages used during the focus group discussions (French, Ditammari, and Waama), supplemented by the field notes. Segments originally spoken in Ditammari or Waama mainly food names and idiomatic expressions, were transcribed in the source language and forward‑translated into French by bilingual team members who had served as moderators or note‑takers and who are native speakers of Ditammari and Waama and fluent in French, ensured familiarity with the discussion context and with culturally specific expressions. An independent bilingual assistant then conducted back‑translation into Ditammari or Waama, and the versions were compared with the originals to ensure semantic equivalence and preservation of culturally specific expressions. Discrepancies were discussed collectively and resolved, with final translations validated by the principal investigator (MMH) to guarantee consistency of terminology before coding [30,31].

Following this verification, two researchers independently coded selected transcripts using the “hybrid deductive-inductive coding approach” [32]. This approach has been used because it allowed us to apply a structured framework (Turner et al.’s food environment framework [10]) while remaining open to emergent themes from students’ narratives, thereby ensuring both theoretical grounding and contextual sensitivity. In To ensure intercoder reliability, two researchers independently coded a subset of transcripts using MAXQDA software (version 24). The independently coded transcripts were then compared, and discrepancies were discussed until consensus was reached. During the initial rounds of independent coding, both researchers moved back and forth between the transcripts and the evolving codebook, refining code definitions, merging conceptually overlapping codes, splitting codes that were found to encompass distinct meanings, and adding new inductive codes as they emerged from the data [33]. Coding discrepancies were resolved through consensus discussions between the two coders, with unresolved cases referred to a third senior researcher for arbitration; the outcomes of these discussions were used to further refine the codebook [34]. This process was repeated across successive subsets of transcripts until no substantive modifications to the codebook were required and no new codes were emerging, indicating that both thematic saturation and codebook stability had been reached. Once the codebook was finalized, all transcripts including those coded during the earlier iterative rounds were fully recoded against the final codebook by one researcher to ensure that the entire dataset was analyzed under a single, consistent version of the coding framework. The second coder conducted periodic consistency checks on a randomly selected proportion of these recoded transcripts, and any remaining discrepancies were resolved through discussion. This iterative recoding step was undertaken to avoid the risk that transcripts coded during earlier stages, when the codebook was still evolving, would be analyzed under an earlier and less refined version of the framework than transcripts coded later [34]. Responses to specific questions were grouped and analyzed to identify emerging themes and sub-themes (see Table 2), making full and appropriate use of quotes illustrating the nature of the interactions observed. Following thematic coding, a comparative analysis was conducted to examine similarities and differences between urban and rural school contexts. Although attention was paid to potential variations by school level during the coding process, no meaningful differences emerged between school level students in the themes identified. The results were therefore presented across school levels.Each coded excerpt cited in the text is referenced using the format *(Transcript, school level, gender, location, Pos. X)*. The abbreviation *“Pos.”* stands for *position* and refers to the numbered segment in the transcript generated by *Maxqda software*, indicating the location of the cited passage within the original text. This referencing system ensures traceability of excerpts while maintaining participant anonymity.

**Table 2:** Emerging study themes and sub-themes.

Themes	Sub-themes
Availability	Food availability Fruits and vegetables availability
Accessibility	Fruits and vegetables accessibility
Quality	Quality of foods available
Food preferences and reason for consumption	

### Trustworthiness and reflexivity

Several strategies were employed to enhance the trustworthiness of the findings, guided by the criteria of credibility, dependability, confirmability, and transferability as proposed by [35,36]. Credibility was strengthened through prolonged engagement with participants, the use of pre-tested interview guides reviewed by a multidisciplinary team, and the inclusion of rich participant quotations to support interpretations. Dependability was ensured through systematic documentation of the research process, including detailed field notes and audio recordings of all FGDs, as well as intercoder reliability checks during data analysis [36]. Confirmability was addressed by maintaining an audit trail of coding decisions and by having two researchers independently code a subset of transcripts, with discrepancies resolved through discussion [36]. Transferability is supported by the provision of detailed contextual information about the study setting, participant characteristics, and data collection procedures, enabling readers to assess the applicability of findings to other contexts.

Regarding researcher reflexivity, the principal investigator (MMH) is a female researcher from Benin with prior experience conducting nutrition research but not in the study area. Even though the moderators were from the study area which facilitated rapport with participants, they were trained to maintain a neutral stance during FGDs and to avoid leading questions. Throughout data collection and analysis, the team engaged in reflexive discussions to examine how their backgrounds, assumptions, and disciplinary perspectives might shape the interpretation of findings. Moreover, member checking was conducted with a subset of participants for validation of the main findings. This study was reported in accordance with the Consolidated Criteria for Reporting Qualitative Research (COREQ) checklist. COREQ provides a structured framework for transparent reporting of qualitative studies using interviews and focus groups. Key elements of the guideline including study setting, participant recruitment, researcher reflexivity, and data analysis procedures were explicitly addressed to allow credibility, dependability, confirmability, and transferability of findings [37]

### Ethics and consents

In accordance with the Declaration of Helsinki, the Bioversity-CIAT Alliance Institutional Review Board’s approval (2022-IRB57) was obtained before the data collection. All participants in this study signed a consent form prior to participating. For students under 18, parental permission to participate was obtained, through a signed consent form, after parents had received all relevant information about the study. Participation in the study was entirely voluntary. All the data collected were anonymously analyzed. No personal identifiers were used.

## Results

### Food availability

Students perceived the term ‘food’ as meaning ‘a meal’ and ‘a recipe.’ Diverse food options were available in the school food environment at the time of the study; some dishes were sold within the school grounds, others outside. Students cited more foods available within the Natitingou school food environment than in Boukombe’s, a difference that may be related to contextual urban–rural variations, as discussed further below. The most important common meals available in the two municipalities were cooked rice mixed with beans (commonly called ‘watché’) within schools, and steamed bean dough (commonly called ‘toubani’) sold at local vendors’ (women) stalls (Table 3).

**Table 3:** Foods available inside and outside secondary schools in Boukombe and Natitingou Municipalities, Norther Benin.

Municipality	Location	Foods available
Boukombé	**Around the school**	Toubani°, rice dough, corn dough, cooked rice, dried yam dough, maize porridge, côme*, creole rice†, fonio dough, pounded yam
	**Within the school**	Roselle juice (’bissap’), porridge, sandwich, watché‡, massa§, wheat patty
Natitingou	**Around the school**	Fonio dough, pounded yam, wheat couscous, spaghetti, peanut sauce with fish, tomato sauce, okra sauce, gari dough, maize dough, dried yam dough
	**Within the school**	Dambou‖, tapioca, soybean porridge, rice porridge, wassa wassa¶, watché‡, toubani°

*Côme: fermented maize dough; **†Creole rice**: seasoned rice with pasta; **‡Watché**: cooked rice mixed with beans; **§Massa**: fermented fried corn fritters; **‖Dambou**: cereal-based couscous with leaves; **¶Wassa wassa**: yam couscous; **°Toubani**: steamed bean dough.

In both settings, students perceived the available foods as predominantly starch-based, with limited protein diversity and few vegetable-based dishes.

### Food preferences and reasons for their consumption

Students cited several reasons for their school-food choices (Table 4). Some students consume foods that are available just to satisfy their hunger. However, many students reported consuming meals considered local or traditional. Some foods, even traditional foods, are not usually prepared within the students’ households but are prepared and sold by street food vendors. As one student mentioned:

**Table 4:** Reasons for consumption and food selection criteria.

Reasons for consumption	Reasons for non-consumption	Selection criteria
Familiarity with local/traditional food	Uncertainty about preparation conditions	Vendor hygiene
Perceived respect for hygiene	Perceived lack of vendor hygiene	Health considerations/allergies
Satisfying hunger	High price	Food preference/habit/prohibitions
Food preference	Unpleasant taste	Food price
Good taste	Use of shea butter or pork fat

*“There are many foods available in and out of the school. Rice-based foods are available at school, but ‘Toubani’ is available outside.” (Transcript, upper secondary school interviews, M, Boukombe, Pos. 15−17)*

Some students do not like to purchase meals sold in or around the schools for various reasons, such as concerns about the vendors’ hygiene practices and the high prices. Some recorded considerations were linked to misleading advertising and common opinions that were not independently verified. In this regard, students participating in the FGDs expressed perceptions and concerns, as follow:

*“I don’t like what is on the street. Sometimes they put ‘pretty juice’ on the sorrel flowers juice, but it doesn’t taste good. It tastes like sugar, but is not really sugar.” (Transcript, lower secondary school interview, F, Boukombe, Pos. 9).*

*“There are allegations where some (vendors) use engine oil or pork oil to prepare ‘massa’.” (Transcript, lower secondary school interviews, M, Boukombe, Pos. 9).*

Moreover, students mentioned several food restrictions, which can be linked to family tradition, health issues, and religious beliefs. The most important were related to meats, such as rooster/chicken, duck, monkey meat, catfish, toad, dog, turtledove, rabbit, and pig.

*“It is forbidden to eat rabbit, turtledove, dog, toad, catfish, egg, chicken” (Transcript, lower secondary school interviews, F, Boukombe, Pos. 15−16).*

*“I do not eat dog and pork, it is forbidden by my religion”. (Transcript, upper secondary school interviews, M, Boukombe, Pos. 45)*

*“In certain families, ceremonies must be held before consuming these forbidden foods” (Transcript, lower secondary school interviews, F, Boukombe, Pos. 17−18)*

Some of the restrictions were also linked to age, food allergies, or intolerance.

*“...I do not eat goat meat; it gives me reflux and I often get diarrhea after eating it.” (Transcript, upper secondary school interviews, F, Boukombe, Pos. 45−47)*

*“... I cannot drink milk; my body does not tolerate it.” (Transcript, upper secondary school interviews, M, Boukombe, Pos. 45−47)*

### Fruit and vegetable availability

A range of 10 fruits and 18 vegetables (5 fruit vegetables and 13 leafy vegetables) were listed by students as available in or around the schools. This encompassed fruit and vegetables available in the food outlets close to the school premises and publicly accessible fruit trees (Table 5).

**Table 5:** List of fruit and vegetables available in and around the school premises by priority.

Fruits	Vegetables	
	Fruit vegetables	Leafy vegetables
Cashew apple, baobab fruit, mango, orange, African locust bean fruit, watermelon, pineapple, papaya, banana, wild strawberry	Okra, tomato, pepper, cucumber, carrot	Baobab leaves, okra leaves, bean leaves, African eggplant leaves, jute mallow, moringa leaves, spicy vegetables, sorrel leaves, African basil, amaranth, bitter leaves, dandelion leaves, lettuce

### Fruit and vegetable accessibility and consumption

The study participants considered a healthy diet to be a balanced diet that includes foods for growth, strength, and protection. In this regard, one of them attested:

*“A healthy diet is when you include all the different food groups in a menu. Foods for strength, foods for growth, and foods for protection”. (Transcript, upper secondary school interviews, M, Boukombe, Pos. 48)*

Despite this awareness, participants commonly reported consuming fruit approximately two to three times per week. Three interconnected barriers emerged consistently across both municipalities: seasonal unavailability, high cost, and limited physical access.

Regarding seasonality, participants noted that fruit and vegetable availability fluctuates sharply between rainy and dry seasons.

One respondent stated:

*“I consume two (2) times, if it’s too much it’s three (3). In the rainy season, we eat vegetables regularly, but in the dry season, it’s almost only dried okra that is consumed the most” (Transcript, upper secondary school interviews, F, Boukombe, Pos. 80−82).*

The influence of season is more pronounced in the municipality of Boukombe, where fruits and vegetables are only available in the school environment during market days. As one student put it:

*“Vegetables are only available on market days, and not only are they small, but a delay in visiting the market results in their unavailability, and occasionally they exhibit a yellow discoloration” (Transcript, upper secondary school interviews, F, Boukombe, Pos. 97−99).*

In addition to the availability challenges related to obtaining fruit and vegetables in both municipalities, most students pointed out issues related to hygiene and affordability. Because of their high price, fruits are generally considered inaccessible to students, even by salespeople.

*“In our community, we have a lot of food, vegetables and fruits, but it’s too expensive, the ladies are not often clean, and on top of that, they insult us” (Transcript, upper secondary school interviews, M,F, Natitingou, Pos. 55−58).*

*“When I say to my mother to buy orange for me, she said that it is better for her to use this money to buy fermented seed of African locust bean than buying orange, which is expensive” (Transcript, upper secondary school interviews, F, Boukombe, Pos. 85−88)*

Students mainly access vegetables at home. The scarcity of vegetables and fruit in these municipalities is due to several factors, including limited access to water and poor soil quality. The rare times when vegetables are available on the markets, they are brought from surrounding villages by the farmers and sold in the open-air market.

*“We don’t grow vegetables here in Boukombe because it’s hard to find water. It’s the women from other villages who bring them on market day, and it’s expensive” (Transcript, upper secondary school interviews, M, Boukombe, Pos. 66−68).*

*“On market days, we have to get to the market early to get them (fruit and vegetables) at affordable prices.” (Transcript, upper secondary school interviews, F, Boukombe, Pos. 73−74).*

In the municipality of Boukombe, there is no food outlet within the 1 km study radius offering vegetable-based dishes. Food promotion was virtually absent, with only rare exceptions for fonio dough, beer, ‘tchoukoutou’ (local beer), and occasionally watermelon when in season.

### Preparation and sanitary quality of foods available

Beyond availability and affordability, the perceived quality and safety of school meals emerged as a significant concern. Participants expressed concerns about food hygiene and poor taste of meals sold in school canteens, as well as inadequate infrastructure

*“The canteen food sometimes has a bad smell, and the water tap is often closed, so we cannot wash our hands or drink.” (Transcript, lower secondary school interviews, F, Boukombe, Pos. 43−45)*

These cumulative concerns: limited variety, poor hygiene, and lack of basic amenities led several participants to characterize their school food environment as inadequate for supporting healthy eating:

*“Our environment is very, very poor in terms of food in general”. (Transcript, upper secondary school interviews, M, Boukombe, Pos. 84−85)*

To improve the quality of the food environment, students described the quality and safety of food available in their environment.

*“We need foods that are well prepared and that don’t give you diarrhea” (Transcript, upper secondary school interviews, M, Boukombe, Pos. 88).*

### Strategies identified by students to improve the availability and accessibility of fruit and vegetables

Students identify several strategies that need to be implemented from their perspectives to improve their nutrition in terms of availability, accessibility, and quality. These include raising awareness about the importance of fruit and vegetables in students’ diets and improving hygiene in all its forms. They also said authorities should organize cooking demonstrations for cooks and food vendors to preserve the nutritional quality of food. They should support establishing school gardens and orchards to facilitate access to fruit and vegetables.

One student suggested:

*“We have to promote and help women who are currently unemployed in creating gardens, with the objective of reducing vegetables prices on the market and increasing the availability.” (Transcript, lower secondary school interviews, M, Boukombe, Pos. 59-60).*

## Discussion

This study examines the perceived impact of school food environments on the diversity and quality of students’ diets in two municipalities in northern Benin. By doing so, it aims to better understand how the availability of food inside and around schools can influence young people’s food choices in this region, which is heavily affected by food insecurity and malnutrition. The results reinforce and extend existing food environment frameworks by suggesting that adolescents’ food choices are shaped not only by physical availability but also by perceived desirability, affordability, and sociocultural meaning. This interplay between external and personal dimensions could be particularly pronounced in food-insecure secondary school settings, where constrained options amplify the influence of taste, economic considerations and health issues. A deep analysis of these factors provides a rich perspective on the eating behaviors of young people in a school setting. More specifically, the present findings confirm several dimensions of Turner et al.’s framework [10], particularly the roles of food availability, affordability, and vendor practices in shaping the external food environment. However, the findings also refine this framework by demonstrating the salience of the perceived dimension in food-insecure secondary school contexts, where cultural food taboos, trust in food vendors, and religious dietary restrictions operate as additional filters through which students interpret and navigate their food environment. Turner et al.’s framework [10] conceptually distinguishes the external food environment (objectively present features such as food outlet density, product range, and physical proximity) from the personal food environment (what individuals perceive as accessible, affordable, and desirable). This distinction has firm empirical grounding: a systematic review of the food environment and diet literature found that perceived measures of the food environment were more consistently associated with dietary outcomes than objectively measured, GIS-based indicators, and proposed that perception captures dimensions of food access such as trust, quality, and desirability that spatial audits alone cannot [38]. Building on this, studies pairing perceived and objective measures of the same food environment have repeatedly reported only low-to-moderate agreement between the two, with the discordance itself, rather than either measure alone, predicting dietary and weight outcomes [39,40]. Among adolescents specifically, a qualitative study of school food environments in Ireland similarly found that reliance on objectively mapped food outlets around schools obscured how adolescents actually perceived and used their environment, and called for adolescent-centred, subjective approaches to complement spatial measures [41]. The present study extends this evidence base to a food-insecure sub-Saharan African setting, where, to our knowledge, adolescents’ perceptions of the school food environment have rarely been documented empirically. Taken together, these findings support a central theoretical contribution of this study: adolescents’ dietary practices could be associated with the objective presence of foods in their environment and by the perceptual, interpretive, and evaluative processes through which that presence is filtered, thereby positioning perception as an independent dimension influencing food choice rather than a passive reflection of objective availability. This suggests that existing food environment frameworks, which were largely developed and tested in high-income settings, may require adaptation to capture the sociocultural complexities of food decision-making in low-resource sub-Saharan African contexts.

### Availability

The two study sites have similar culinary traditions with meals characterized by a predominance of starchy staples such as maize, yam, beans, and fonio (also known as ‘hungry rice’). However, the urban area surrounding Natitingou secondary school offered a greater diversity of foods. This observation reflects a common trend in rural areas, especially in low- and middle-income countries, where positive links exist between urbanization levels and the presence of large food vendors [10]. In rural areas, access to diverse food options is limited by economic and geographical factors, for example, road infrastructure quality [42]. In fact, urbanization, combined with higher incomes, promotes a greater variety of and demand for food options [43]. Most of the time, foods are transported from rural areas to urban centers, sometimes increasing their availability in these locations even beyond production periods. Beyond geographic and socioeconomic disparities, differences in local governance, market infrastructure, and enforcement of food hygiene regulations may also contribute to the observed urban–rural contrasts. Variations in municipal oversight and regulatory implementation could influence both the diversity and safety of foods available in and around schools.

Seasonal factors such as poor rainfall, limited water access, and degraded soil quality were identified as key contributors to the lack of fruits and vegetables, especially during the dry season. Seasonal barriers appeared more pronounced in Boukombe (rural), where students directly linked food availability to local agricultural cycles and harvest periods. During the dry season, fruits and vegetables were described as scarce and expensive, reflecting production constraints typical of rural agrarian settings. In contrast, although students in Natitingou (urban) also reported seasonal price increases, food availability was perceived as relatively more stable due to greater market integration and supply from multiple sources. This suggests that seasonality operates differently across rural and urban school food environments, with stronger production-related constraints in rural contexts. High cost was also highlighted. Similar challenges regarding seasonal availability, physical accessibility, as well as economic barriers have been noted by previous studies [44,45]. Although seasonal and economic barriers are rooted in broader structural constraints, they are not entirely immutable. Targeted support for small-scale dry-season horticulture, improved storage and preservation of perishable produce, and strengthened local market linkages could help reduce seasonal shortages and price fluctuations. Such measures may improve the availability of fruits and vegetables even in resource-constrained rural settings. Moreover, issues such as poor hygiene and high prices deterred some students from consuming local foods. Beyond the nutritional quality of the foods, food safety was also highlighted by the students as a key aspect of healthy diets, since poor hygiene can affect overall food quality and can lead to students’ illness [46].

### Accessibility

In both settings, students perceived their food environment as unhealthy. Many students reported limited access to healthier food options, with accessibility restricting their ability to consume healthy and nutritious foods. Within Turner et al.’s framework [10], this finding illustrates the importance of distinguishing between the external food environment (what is physically present) and the personal food environment (what students perceive as accessible, affordable, and desirable). While students demonstrated a general understanding of the importance of a balanced diet, many struggled to incorporate healthy food options in their daily meals. Due to limited physical and economic accessibility to healthier options, students were reportedly forced to consume what was readily available simply to satisfy their hunger. So, they focused on food quantity to fill their stomachs, rather than food quality reflecting nutritional value. The availability of fruit and vegetables in the schools’ food environment was limited in both settings; hence, little opportunity for students to find and eat fruit and vegetables at school [47]. It is known that easier access to foods with high nutritional value is linked to healthier eating habits [48]. This result aligns with the study conducted on the same target group in the same area, which showed that these students do not consume enough fruit and vegetables to meet the daily requirement of 400g [5]. Since students spend most of their time at school, studies [49,50] show that inadequate access to nutritious foods in the school food environment was associated with less favorable eating habits and may be linked to increased risk of malnutrition and related health concerns, although causal relationships cannot be established from cross-sectional qualitative data.

### Urban–rural comparison

The findings reveal a consistent pattern of urban-rural differences across multiple dimensions of the school food environment. In terms of food availability, the urban setting (Natitingou) offered a wider diversity of prepared foods. This difference may be related to greater market infrastructure and commercial activity in urban areas, although these factors were not directly assessed in the present study. In terms of seasonal vulnerability, students in the rural setting (Boukombe) reported sharper fluctuations in fruit and vegetable availability tied to local agricultural cycles, whereas the urban setting benefited from supply from multiple sources. Regarding physical access, the rural school environment lacked food outlets offering vegetable-based dishes within the 1 km study radius, while the urban setting, despite its own limitations, provided more food outlet options. However, certain challenges were shared across both settings, including high food prices, hygiene concerns, and a predominance of starch-based meals with limited nutritional diversity. Interpreted through Turner et al.’s framework [10], these differences suggest that external dimensions of the food environment may vary across urban and rural settings. Factors such as vendor density, market integration, and supply chain connectivity could play a role in shaping what foods students can realistically access; however, these factors were not directly measured in the present study and therefore warrant further investigation. These findings suggest that interventions to improve school food environments may need to be tailored to the urban or rural context, with rural settings requiring more attention to local production support and supply chain strengthening, while urban settings may benefit more from vendor regulation and hygiene enforcement.

### Actions needed

Students’ perceptions of the school food environment reflect not only individual and contextual factors within schools, but also the broader political and institutional environment. In Benin, policies such as the National Food and Nutrition Policy (PNAN) and the Integrated School Feeding Programme (PNASI) influence the extent to which healthy food options are promoted or made accessible in school settings. However, the limited visibility of these programs at the secondary school level may partly explain why secondary school students report that food environments are dominated by convenience and affordability, rather than featuring health-oriented choices. Integrating this political dimension is essential, as it highlights the enabling environment within which secondary school students construct their perceptions and practices.

To address all these challenges and promote healthier eating habits, several potential measures could be implemented, within the school environment and beyond, that may help improve students’ access to fruit and vegetables. Drawing directly from the barriers identified in this study, the following recommendations are proposed as potential strategies for consideration, organized by the dimension of the food environment they address:

To improve physical availability, initiatives such as creating school gardens and orchards may represent promising approaches for increasing access to fruit and vegetables, even during off-seasons, and offer valuable food culture learning experiences, serving as educational tools for students [51,52]Promoting preservation methods for fruit and vegetables may also help mitigate the effects of seasonal variability, ensuring year-round availability.

To improve economic accessibility, policymakers and stakeholders could consider fair pricing strategies aimed at making local foods more affordable and appealing to students. Supporting local food production through dry-season horticulture programs could also help reduce market prices by increasing supply.

To improve food quality and safety, supporting food vendors through cooking demonstrations and hygiene training may represent a useful strategy for enhancing food safety practices and potentially improving the appeal of locally prepared and nutrient-rich foods to students [53]. Strengthening oversight of foods sold within school premises could be considered as a potential approach to improving food safety standards.

To address the personal food environment, nutrition education programs could be considered as a potential strategy to raise awareness about the benefits of balanced diets and encourage better food choices, particularly by building on students’ existing knowledge of local food [54].

In terms of implementation sequencing, priority could be given in the short term to low-cost school-level interventions particularly nutrition education and hygiene training for food vendors as these are immediately feasible within existing institutional structures. In the medium to longer term, secondary schools could be integrated into existing national nutrition and food security initiatives, particularly through intersectoral collaboration, vendor regulation, and nutrition education. These measures could collectively improve the food environment in schools, help close nutritional gaps, and foster healthier eating attitudes and behaviors among students and motivate students to advocate for healthier food options in and beyond school, as well as within households. However, these recommendations should be interpreted as policy implications derived from students’ reported experiences and perceptions, rather than as interventions whose effectiveness has been demonstrated by the present study.

Although this study relied exclusively on focus group discussions, the findings provide valuable insight into how adolescents collectively perceive and interpret their school food environments. However, there are some limitations that need to be acknowledged:

First, the study was based on only two secondary schools located within a single region of northern Benin (Atacora department), with a total of 40 participants across four focus group discussions. This limited geographical scope means that the findings may not fully represent adolescents attending schools in other settings within Benin or to other West African countries with different food systems, cultural practices, or school food policies. While the sample size and the number of focus group are consistent with empirical guidance on saturation in focus group research [29], saturation addresses thematic completeness within the studied population rather than transferability across geographical or institutional contexts. The purposive selection of schools in Boukombe and Natitingou was intended to capture urban–rural contrasts. Second, focus group discussions may be subject to social desirability bias, whereby participants provide responses they perceive as socially acceptable rather than reflecting their true perceptions or behaviors. Additionally, the dynamics inherent to focus group settings, including peer influence, conformity pressure, and dominance by more outspoken participants, may have shaped the range and depth of responses obtained. Although techniques such as turn-taking prompts and targeted invitations to quieter participants were employed to mitigate these effects, it is possible that some individual perspectives were underrepresented in the group discussions. Third, the FGDs were conducted in local languages (Ditammari and Waama) and French, transcripts were translated into French for analysis. Although a back-translation procedure was employed and translations were verified by bilingual team members familiar with the study context, some nuances of meaning, culturally specific expressions, or idiomatic language may have been lost or altered during the translation process. The use of an interactive qualitative approach enabled the exploration of shared norms, social dynamics, and context-specific meanings that would be difficult to capture through quantitative or observational measures alone. Future research could focus on a mixed-method designs that combine objective assessments of the school food environment with in-depth qualitative exploration of adolescent perceptions, allowing potential differences between perceived and objectively measured food environments to be examined and quantified. Longitudinal investigations tracking changes in adolescent dietary practices across school years and seasons would clarify whether observed patterns persist over time and how seasonal fluctuations shape long-term dietary habits. Finally, intervention-based research evaluating school-level improvements is needed to test whether addressing the barriers identified in this study translates into measurable improvements in adolescent diets. While the present manuscript emphasizes the perceived dimension of the school food environment, the objective assessment of food availability, diversity, and vendor practices is addressed in a complementary study currently under preparation. Together, these strands of evidence contribute to a more comprehensive understanding of secondary school food environments in Northern Benin.

## Conclusions

This qualitative study provides important insights into how secondary school students in Boukombe (rural) and Natitingou (urban) perceive and navigate their school food environments. It reveals that limited dietary diversity, combined with economic, seasonal, cultural, and safety constraints, shapes students’ food choices in ways that extend far beyond simple availability. The findings also highlight meaningful urban-rural disparities, with rural students facing greater structural constraints, while challenges related to affordability and hygiene cut across both contexts. Together, these findings emphasize that improving school food environments requires more than increasing food availability; it demands integrated, context-sensitive strategies that account for students lived realities. They further highlight the importance of considering students’ perspectives when designing policies and programs aimed at improving school food environments. Future research integrating subjective and objective assessments could provide a more comprehensive understanding of the factors influencing adolescents’ food choices in food-insecure settings. Although this study is context-specific, it underscores a broader implication: creating healthier school food environments in food-insecure settings will depend on participatory approaches that make nutritious foods not only accessible, but also affordable, safe, and desirable.

### Paper context

- Main findings: Students viewed their school food environments as unhealthy and unhygienic, where foods are mostly energy-dense and sugar-rich, with few or no fruits and vegetables.
- **Added knowledge:** Provides the first qualitative evidence on secondary school students’ views of school food environments in Northern Benin and fills a key knowledge gap on barriers to healthy eating in low-resource secondary school settings**.**
- **Global health impact for policy and action:** Strengthening school food environments including supportive food policies in low- and middle-income countries is vital to ensure safe, nutritious and affordable food that promotes healthier secondary school students’ diets, while also including secondary school students’ perspectives to improve the design, acceptability and uptake of interventions.
